# Prevalence of tuberculous lymphadenitis in Gondar University Hospital, Northwest Ethiopia

**DOI:** 10.1186/1471-2458-13-435

**Published:** 2013-05-03

**Authors:** Dagnachew Muluye, Belete Biadgo, Eden W/Gerima, Andebet Ambachew

**Affiliations:** 1 School of Biomedical and Laboratory Sciences, College of Medicine and Health Sciences, University of Gondar, P.O. Box. 196, Gondar, Ethiopia

**Keywords:** Tuberculous lymphadenitis, Fine needle aspiration cytology, Ethiopia

## Abstract

**Background:**

Tuberculous is the leading cause of death worldwide with a large number of deaths occurring in developing countries. Tuberculous lymphadenitis is among the most common presentations of extra pulmonary tuberculous. This study attempts to determine the magnitude of tuberculous lymphadenitis from patients with lymph node aspirate in Gondar University Hospital, Northwest Ethiopia.

**Methods:**

Retrospective study was conducted. Data were collected from registration book of Gondar university Hospital pathology laboratory after checking the completeness of patient’s necessary information like age, sex and fine needle aspiration cytology results. Data were entered and analyzed using SPSS version 16 statistical package. Chi-square test was done to determine associations.

**Result:**

A total of 3,440 lymph node aspirates were examined using fine needle aspiration cytology. Of these, 2,392 (69.5%) cases were found to have tuberculous lymphadenitis. Male 1647(47.9%) to female 1793(52.1%) ratio of all study subjects were 0.9:1. Females (54.1%) were more affected than males (45.9%). Age, sex and site of aspiration were found to be statistically associated with tuberculous lymphadenitis (p-value < 0.001). The age group of 15–24 years had the highest prevalence of tuberculous lymphadenitis followed by those of 25–34 years old. The most affected sites were cervical lymph nodes (47.5%) followed by auxiliary (19.4%) and submandibular (12.9%) lymph node regions. None of the records documented the HIV status of subjects.

**Conclusion:**

The prevalence of tuberculous from lymph node aspirate was found to be higher involving the frequently affected site of cervical lymph node. The HIV status of patients with all forms of tuberculous should have to be checked and documented. Further prospective and advanced studies are recommended to determine the specific etiologic agents and contributing factors.

## Background

Tuberculous (TB) is a chronic bacterial disease caused by a slightly curved non motile, aerobic, non-capsulated and non-spore forming strains of mycobacteria usually *Mycobacterium tuberculous*[1]. It is a major public health problem in the world and 1/3 of world’s population is infected predominantly in developing countries [2]. The magnitude of TB is higher in the developing world due to various factors including malnutrition, different causes of immune suppression, dual HIV-TB epidemic and increasing causes of drug resistant TB (Multi drug resistant TB (MDRTB), Extreme drug resistant TB (X-DRTB)) [3]. Ethiopia ranks 7th in the list of 22 high burdens countries severely affected by tuberculous [4]. As per WHO global TB report of 2012, the estimated incidence of all forms of TB in Ethiopia was 220/100,000 population [5] and smear positive cases was 63/100,000 population [6].

Extra pulmonary tuberculous (EPTB) affects different organs of human body where tuberculous lymphadenitis (TBLN) is the most common manifestations of all EPTB [7-10]. The most commonly involved lymph nodes were cervical, axilliary, inguinal, abdominal and supra clavicular sites. Cervical lymph nodes are the most commonly affected group of nodes [11]. Tuberculous that affects cervical lymph nodes represents about 50% of EPTB even though it could vary in different areas [12]. A number of studies showed higher proportion of tuberculous lymphadenitis among patients in different areas [13-16]. In a study conducted in the rural part of Ethiopia, 72.8% cases of TBLN were found [17] and the cervical region was the most affected site (74.2%) followed by the axillaries and inguinal lymph node regions, 20.3% and 4.3%, respectively [18].

In Ethiopia, there are only few reports concerning TBLN. Particularly in Gondar there is no data that shows the magnitude of TBLN. This study attempts to assess the magnitude of TBLN and provide baseline information for health professionals and other concerned bodies. Therefore, the aim of the study was to assess magnitude of TBLN from patients with lymph node aspirate in Gondar University Hospital.

## Methods

A retrospective study of seven years period from January, 2003 to January, 2007 and January, 2010 to January 2011 was carried out in Gondar University Hospital pathology laboratory registration book. Gondar is found in North West part of Ethiopia, with in the Amhara regional state at about 748 and 175 kilometers away from the capital Addis Ababa and Bahir Dar respectively. The population of the town is about 206,987 as stated in central statistical agency (CSA) of Ethiopia, 2007.

The study populations were all patients with lymph node aspirate in Gondar University Hospital. The study subjects were all patients with lymph node aspirate between January, 2003 and January, 2007 and January, 2010 to January, 2011. All patients with lymph node aspirate between January, 2003 to January, 2007 and January, 2010 to January, 2011 were included in the study by collecting data from the registration book. Records with incomplete data and demographic characteristics were excluded. TBLN diagnosis by cytology was made by examination of the presence of caseous necrosis, epitheloid cell granulomas, multi nucleated giant cells, degenerated epithelioid cell with neutrophil and hetrogeneous lymphoid population plus granulomatous features.

Data were collected by investigators from registration book of Gondar university Hospital pathology laboratory after checking the completeness of patient’s necessary information like age, sex and fine needle aspiration cytology results. Data were analyzed using SPSS version 16 statistical package. Data were summarized using descriptive statistics. Association was done using Chi-square test and P-value <0.05 were considered statistically significant.

### Ethical consideration

Data were collected after ethical clearance obtained from the School of Biomedical and Laboratory Sciences, College of Medicine and Health Science, University of Gondar. After discussing the purpose and aim of the study, permission was obtained from the Head of Gondar University Hospital pathology laboratory before the data collection.

## Result

**Figure 1:**
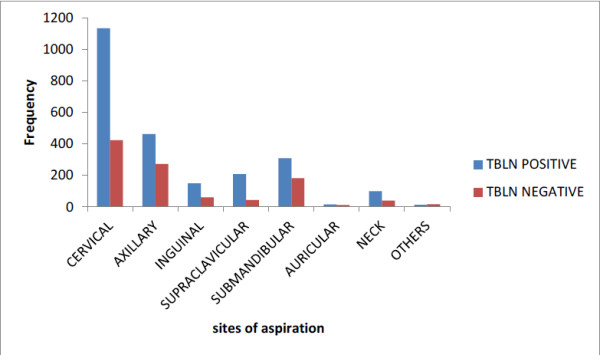
Prevalence of tuberculous lymphadenitis by Site of aspiration among patients with lymph node aspirate in Gondar University Hospital from January, 2003– January, 2007 and January, 2010– January, 2011 G.C.

A seven year period retrospective study was carried out to determine the prevalence of tuberculous lymphadenitis among patients examined by FNAC at Gondar University Hospital pathology laboratory. A total of 3,440 lymph node aspirates were examined. Out of these, 2,392 (69.5%) were found to have cytological findings suggestive of TBLN and 1,048 (30.5%) cases were diagnosed as reactive lymphadenitis. Male 1,647 (47.9%) to female 1,793 (52.1%) ratio of all study subjects were 0.9:1. Tuberculous lymphadenitis was found among 1,098 (45.9%) males and 1294 (54.1%) females with an overall prevalence of 69.5% (Table 1). Females are relatively more affected than males. Higher proportion of tuberculous lymphadenitis were found among the age group of 15–24 years (28.5%) and 25–34 years (27.6%) (Table 1). The most affected sites were cervical lymph nodes 1135 (47.5%); axilliary 463 (19.4%) and submandibular 308 (12.9%). The least affected lymph node sites were the auricular, neck and other sites with 6%, 4.2% and 0.5% respectively (Figure 1).

**Table 1:** Distribution of patients with tuberculous lymphadenitis by sex, age and site of aspiration in Gondar University Hospital from January, 2003– January, 2007 and January, 2010– January, 2011 G.C.

**Variables**	**Tuberculous lymphadenitis**		**X^2^**	**P value**
	**Positive (%)**	**Negative (%)**		
**Age**			10.402	**<0.001**
<5	108(4.5)	113 (10.4)		
5-14	296 (12.6)	221 (21.1)		
15-24	682 (28.5)	255 (24.3)		
25-34	660 (27.6)	246 (23.5)		
35-44	347 (14.5)	119 (11.4)		
≥45	299 (12.5)	94 (9.0)		
**Sex**			12.271	**<0.001**
Male	1098 (45.9)	549 (52.4)		
Female	1294 (54.1)	499 (47.6)		
**Site of aspiration**			65.749	**<0.001**
Cervical	1135 (47.4)	423 (40.4)		
Axillary	463 19.4)	272 (28.0)		
Inguinal	150 (6.3)	60 (5.7)		
Supraclavicular	208 (8.7)	43 (4.1)		
Submandibular	308 (12.9)	182 (17.4)		
Auricular	15 (0.6)	12 (1.1)		
Neck	100 (4.2)	40 (3.8)		
Others	13 (0.5)	16 (1.5)		

**Figure 2:**
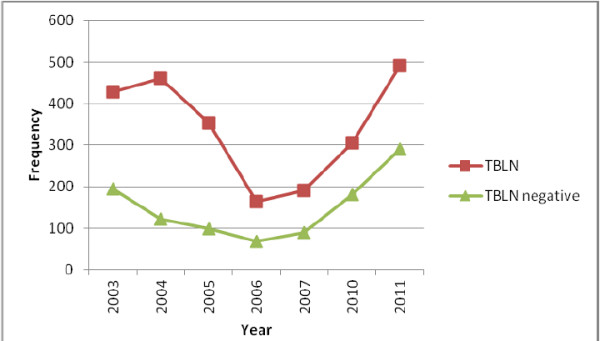
Trend prevalence of tuberculous lymphadenitis among patients with lymph node aspirate in Gondar University Hospital from January, 2003– January, 2007 and January, 2010– January, 2011 G.C.

The trend prevalence of TBLN over the years is fluctuating from 234 to 781 cases. The trend has continued with no great difference in 2003 and 2004 with a total of 623 and 582 TBLN cases but lower in the year 2005 and 2006 that was 453 and 234 cases respectively. In the year 2007, 2010 and 2011, the occurrence rises to 280, 487 and 781 cases respectively (Figure 2).

Age, sex and Site of aspiration were found to be significantly associated with TBLN. The age group of 15–24 years had the highest prevalence 682 (28.5%) followed by those of 25–34 years with 660 (27.6%) and 35–44 years with 347 (14.5%) and >45 years with 299 (12.5%) (P-value < 0.001). Tuberculous lymphadenitis was higher in females than males with statistically significant difference (p-value < 0.001). The most affected sites were cervical lymph nodes, axillary and submandibular with statistically significant difference (p-value < 0.001) (Table 1). None of the records documented the HIV status of patients.

## Discussion

The prevalence of tuberculous lymphadenitis has been reported to be higher in developing countries like Ethiopia compared to developed countries [2]. In this study, the prevalence of TBLN was 69.5% which is identical with other study done in Tanzania with prevalence of 69.5% [14]. Other studies in Israel, Ethiopia and India showed similar figure with prevalence of 70%, 72.8% and 73.75% [17,19,20] but to some extent higher in another Indian study with prevalence of 83% [11]. The higher prevalence in this Indian study could be due to the sample size and the time of study where awareness of TB was poor years ago. In contrast to this study, the prevalence of TBLN was lower in Nigeria (24.45%), Pakistan (44%) and India (62%) [13,15,21]. This difference could be due to the different study methods used where small sample size was used in Pakistan and in India. In addition, these studies were onetime studies while our study is retrospective study.

The age profile of patients with TBLN showed involvement of younger patients with 15–24 years old being affected accounting 28.5% followed by 25–34 years old accounting 27.6% of the cases. This finding is similar to other studies where younger than 30 years old are the commonest age group affected by this disease [18]. Similar study in Nigeria showed the age group of 10–19 years old with highest prevalence (28.1%) and 20–29 years with 21.8% which is consistent with our study but the magnitude of the age group 0–9 years has prevalence of 26.3% which is higher than our study [15]. Male to female ratio of our study is found to be 0.9:1 in which females being highly affected than males with no obvious preponderance in Israel, Pakistan, Nigeria, Ethiopia [11,15,18,19]. Cervical lymph node was found to be the most commonly affected site (47.5%) compared to axillaries and other affected sites. This finding is in agreement with the previous study done in Ethiopia where cervical regions (74.2%) being mostly affected site followed by axillary (20.3%) and inguinal regions (4.3%) [18] and the study in India and Nigeria were also revealed similar manner [15,21]. Historically, tuberculous cervical lymphadenitis has been more common in children and young adults [22].

The trend prevalence of TBLN cases in this study was variable over the years revealing increment in the recent years. The variation in patterns of prevalence of TBLN might be due to TB-HIV co-infection, other immune compromization (chronic diseases) that causes immune suppression and increased risk of developing tuberculous, awareness of people about early diagnosis and treatment of TB patients. Especially in 2003 and 2004 before the era of anti retroviral therapy, the numbers of cases were higher compared to 2005 and 2006. The reason for decreased prevalence in the two consecutive years could be due to lack of service expansion in the hospital hence patients were appointed for more than a month and cannot come back to the hospital. In 2007, 2010 and 2011, there was slight increment in number of cases. This might be due to the increased awareness of community about tuberculous and get diagnosed than the previous years. Increased cases of malnutrition, chronic diseases and MDR-TB could also exaggerate the rate of TBLN in these recent years. The other reason could be increased patient health seeking behavior, service expansion where patients were not appointed not more than a week, well equipped laboratory service and increased number of pathologist by which the quality of the service increased the case detection rate as well as the number of patients compared to the previous years. Previously there was no or one off and on type of pathologist but recently there are at least three actively working pathologists which might contribute for increased case detection rate.

This study tried to assess yet untouched area in the study site. It is very valuable and informative which could give insights for health professionals and policy makers to address the problem. The HIV status of patients with TB (prior emphasis for EPTB) should have to be checked and documented. We couldn’t able to include other socio demographic variables which has contribution for tuberculous because of incomplete registration. Lack of the two years data in between due to fire accident on pathology laboratory was also the dare for generalization to the overall trend prevalence.

## Conclusion

The prevalence of TBLN in patients with lymph node aspirate in Gondar University Hospital is high. Among all lymph node sites, cervical region was the predominantly affected site compared to other sites. Female patients were more affected than males. It is important for pathologists to be conscious of tuberculous cases whenever they encounter enlarged lymph node region. The HIV status of patients with TB (prior emphasis for EPTB) should have to be checked and documented. Further prospective and advanced studies are recommended to determine the specific etiologic agents and contributing factors of TBLN in the study area.

## Competing interests

The authors declare that they have no competing interests.

## Authors’ contributions

DM: conceived the study, undertook statistical analysis and drafted the manuscript. BB, EW and AA: initiated the study and made major contributions to the data collection and statistical analysis. All authors contributed to the writing of the manuscript and approved the submitted version of the manuscript.

## Pre-publication history

The pre-publication history for this paper can be accessed here:

http://www.biomedcentral.com/1471-2458/13/435/prepub
